# Individualized high-resolution analysis to categorize diverse learning and memory deficits in tau rTg4510 mice exposed to low-intensity blast

**DOI:** 10.3389/fncel.2024.1397046

**Published:** 2024-06-13

**Authors:** Amitai Zuckerman, Heather R. Siedhoff, Ashley Balderrama, Runting Li, Grace Y. Sun, David X. Cifu, Ibolja Cernak, Jiankun Cui, Zezong Gu

**Affiliations:** ^1^Department of Pathology and Anatomical Sciences, University of Missouri School of Medicine, Columbia, MO, United States; ^2^Harry S. Truman Memorial Veterans’ Hospital Research Service, Columbia, MO, United States; ^3^Biochemistry Department, University of Missouri, Columbia, MO, United States; ^4^Department of Physical Medicine and Rehabilitation, Virginia Commonwealth University School of Medicine, Richmond, VA, United States; ^5^Thomas F. Frist, Jr. College of Medicine, Belmont University, Nashville, TN, United States

**Keywords:** mild traumatic brain injury, low intensity blast, tau rTg4510 mice, CognitionWall, heterogeneous learning patterns, clinical translation value

## Abstract

Mild traumatic brain injury (mTBI) resulting from low-intensity blast (LIB) exposure in military and civilian individuals is linked to enduring behavioral and cognitive abnormalities. These injuries can serve as confounding risk factors for the development of neurodegenerative disorders, including Alzheimer’s disease-related dementias (ADRD). Recent animal studies have demonstrated LIB-induced brain damage at the molecular and nanoscale levels. Nevertheless, the mechanisms linking these damages to cognitive abnormalities are unresolved. Challenges preventing the translation of preclinical studies into meaningful findings in “real-world clinics” encompass the heterogeneity observed between different species and strains, variable time durations of the tests, quantification of dosing effects and differing approaches to data analysis. Moreover, while behavioral tests in most pre-clinical studies are conducted at the group level, clinical tests are predominantly assessed on an individual basis. In this investigation, we advanced a high-resolution and sensitive method utilizing the CognitionWall test system and applying reversal learning data to the Boltzmann fitting curves. A flow chart was developed that enable categorizing individual mouse to different levels of learning deficits and patterns. In this study, rTg4510 mice, which represent a neuropathology model due to elevated levels of tau P301L, together with the non-carrier genotype were exposed to LIB. Results revealed distinct and intricate patterns of learning deficits and patterns within each group and in relation to blast exposure. With the current findings, it is possible to establish connections between mice with specific cognitive deficits to molecular changes. This approach can enhance the translational value of preclinical findings and also allow for future development of a precision clinical treatment plan for ameliorating neurologic damage of individuals with mTBI.

## 1 Introduction

Exposure to low-intensity blast (LIB) has been shown to cause mild traumatic brain injury (mTBI) in military and civilian settings. In most situations, subjects exposed to LIB do not show overt physical impairments, and neuroimaging is often unable to detect abnormalities (Cernak, 2017). Nevertheless, preclinical studies with animal models have unveiled effects of LIB exposure causing molecular and nanoscale changes in brain. Ultrastructure changes, such as altered mitochondria and synaptic structures and changes in proteosomes and biochemical pathways, are indications suggesting LIB-induced cellular and molecular injuries (Chen et al., 2018, 2022; Song et al., 2018a, 2019; Konan et al., 2019). These “invisible injuries” have been regarded as the underlying causes leading to neurologic (e.g., dizziness, spatial disorientation, vision issues), somatic (e.g., headache, sleep disturbance, chronic pain) and mental health (e.g., depression, anxiety, post-traumatic stress disorder [PTSD]) symptoms. More importantly, mTBI due to blast has been regarded as a contributing factor for cognitive (e.g., poor memory, decreased concentration), and neurodegenerative disorders [e.g., Alzheimer’s disease-related dementias (ADRD), tauopathies, frontotemporal dementia (FTD), chronic traumatic encephalopathy (CTE)] (Elder et al., 2019; Robinson-Freeman et al., 2020; Siedhoff et al., 2021; Flavin et al., 2023).

For many years, there is the evolving concept that each type of clinical dementia is represented by a different type of brain pathology. However, more recent findings from preclinical and clinical studies show a shift in this concept due to a high complexity of these pathologies. The report by the ADRD 2022 Summit summarized this shift and suggested that “*there is not a one-to-one relationship between the type of brain pathology present and the clinically assigned dementia diagnosis in most individuals*” (Rost, n.d.). In fact, recommendations by the ADRD Summit and its Post-TBI AD/ADRD sub-committee (Dams-O’Connor et al., 2023) included: (i) “*generate research results that are more generalizable to the real-world; (ii) “maximize clinical translatability in the study of TBI-AD/ADRD”; and (iii) “basic and translational research to elucidate the mechanistic pathways, development, and progression of post-TBI AD/ADRD neuropathologies to better understand clinical symptom expression*” (Rost, n.d.).

Over the past decade, numerous animal models have been crafted to gain a deeper understanding of the distinctive aspects of brain damage induced by blast, aiming to replicate “real-world scenarios” of mTBI (Cernak et al., 2011; Rubovitch et al., 2011, 2017; Zuckerman et al., 2017, 2019; Song et al., 2018a,b; Hoffman et al., 2020; Chen et al., 2022). Research involving live animals has broadened our comprehension of impairments and abnormalities in the brain due to blast across various levels (molecular, cellular, and behavioral) and the progression observed at different post-injury timepoints (Rubovitch et al., 2011, 2017; Zuckerman et al., 2017, 2019; Chen et al., 2018, 2022; Song et al., 2018a,c, 2019; Konan et al., 2019; Herzog et al., 2020; Hoffman et al., 2020; Siedhoff et al., 2022). In individuals affected by blast injury, cognitive disorders, including difficulties in memory, concentration, and deficits in multitasking, can manifest shortly after the injury, and these disorders may persist over a long term, posing a chronic burden for both patients and their families.

Despite several studies utilizing mouse models to demonstrate LIB-induced ultrastructural and molecular changes (Rubovitch et al., 2011, 2017; Chen et al., 2018, 2022; Song et al., 2018a,c, 2019; Konan et al., 2019; Siedhoff et al., 2022), the mechanisms connecting these alterations to specific behavioral outcomes remain unclear. In preclinical studies, the most frequently used assessments for learning and memory include the novel object recognition (NOR), Morris Water Maze (MWM) and the Barnes Maze tests. These tests initially evaluate the animal’s ability to learn and memory using spatial cues to locate an “exit point.” Then, during the second phase, they assess the animal’s cognitive flexibility by changing the location of the “exit point” (Song et al., 2018a; Arulsamy et al., 2019; Leconte et al., 2020; Chen et al., 2022). Although these tests significantly contribute to the understanding of learning and memory processes in both injured and non-injured animals, they have the several limitations including: (i) The learning and memory abilities differ between species, strains, and ages of the animals. (ii) These tests are based on repeated short (1–2 min) sessions during which the animals are placed in the testing environment and are conducted only during the light or dark phase, and not both. (iii) Unlike clinical studies, where evaluations are conducted at an individual level, most preclinical tests are statistically evaluated at the group level (Rubovitch et al., 2011, 2017; Zuckerman et al., 2017, 2019; Chen et al., 2018, 2022; Song et al., 2018a,c, 2019; Konan et al., 2019; Herzog et al., 2020; Hoffman et al., 2020; Siedhoff et al., 2022). Consequently, variations in the analytical approach may constrain the translation of preclinical findings into practical applications in clinical settings.

Considering these apparent limitations and the recommendations of the ADRD Summit 2022 Report (Dams-O’Connor et al., 2023), a novel assessment method utilizing the CognitionWall test (Remmelink et al., 2016; Logan et al., 2018; Chen et al., 2022) has been devised that enables the evaluation of learning ability and general behavior in an individual mouse over an extended period encompassing both light and dark phases. This method generates data by recording the number of entries into the CognitionWall each hour and utilizing this information to construct individual Boltzmann curves. In this study, we used the transgenic (Tg) rTg4510 mice, a murine model with repressible form of human tau containing the P301L mutation (hTau_P301L_). This model, recapitulating tauopathies and neurodegeneration, has been linked with familial frontotemporal dementia (FTD) (Bailey et al., 2014; Gamache et al., 2019). The outcomes demonstrate high complexity and variations across different groups with respect to genotypes and exposure to LIB. Following this, a flow chart is devised for the evaluation and processing of data, leading to the categorization of individuals within the group based on distinct learning patterns and levels of learning deficits.

## 2 Materials and methods

### 2.1 Animals

All animal experiments were performed in a blinded manner and in accordance with the University of Missouri approved protocols for the Care and Use of Laboratory Animals and the Animal Research using the Reporting of *In Vivo* Experiments (ARRIVE) guidelines. This study included a total of 65 male mice at the aged 6-weeks-old upon arrival. This mouse strain was derived from two genotypes, hemizygous (HEMI) for Tg(Camk2a-tTA)1Mmay and Fgf14Tg(tetO-MAPT*P301L)4510Kha/J (Jackson Laboratories, Bar Harbor, ME, United States; Strain #/RRID: IMSR_JAX:024854) and has the common name as rTg4510, a murine model of tauopathies. The HEMI mice were chosen for their feature of expressing high levels of hTau_P301L_ in their forebrains. From the 65 mice, 32 mice were from the HEMI group and the remaining 33 mice were non-carrier (NCAR; RRID: IMSR_JAX:019019) generated by breeding with C57BL/6 J females, recommended by and purchased from the Jackson Laboratory. Mice were housed with a 12-h light/dark cycle in home-cages containing bedding, and with *ad libitum* access to food and water. Mice were used in a series of 3 independent experiments (20–23 mice per experiment).

### 2.2 Exposure to open-field low-intensity blast

Mice were exposed to open-field blast at 2 months of age. Open-field LIB exposures were conducted at the Missouri University of Science & Technology as previously reported (Chen et al., 2018, 2022; Song et al., 2018a,b,c; Konan et al., 2019; Song et al., 2019; Clausen et al., 2021; Rutter et al., 2021; Siedhoff et al., 2022). Animals were randomly divided into four groups: (NCAR-Sham: *n* = 15, NCAR-Blast: *n* = 18, HEMI-Sham: *n* = 13, and HEMI-Blast: *n* = 19). Prior to the experiment, each mouse was anesthetized by injection I.P. with 10 μL/g bodyweight of ketamine/xylazine mixture (12.5 mg/mL ketamine and 0.625 mg/mL xylazine). Mice from the blast groups (NCAR-Blast & HEMI-Blast) were placed in an upright position in 3D-printed chairs made of carbon reinforced nylon (Nylon X, Matterhackers) (Jackson et al., 2024a). This position was held using elastic mesh bands to restrain the head and body movements. The mouse holders were placed on a platform at 3 meters from the site of detonation with a 350 g C4 explosive for a single blast exposure (with peak overpressure of 46.6 kPa). Following LIB exposure and after fully awakened from anesthesia, mice were returned to their original cages. No mortality among these mice occurred from the blast procedure in this study. Mice were monitored during the entire anesthesia time, and 15–30 min after fully awakened from anesthesia. Sham mice were treated as the blast mice, but did not expose to the LIB.

### 2.3 Automated assessments of learning in a home-cage environment

Based on results of our previous study (Song et al., 2018a), assessment of learning abilities and cognitive flexibility, was initiated 15- or 16-days post-exposure that belong to the early subacute phase using the PhenoTyper (Model 3,000, Noldus Information Technology, The Netherlands), an automated home-cage monitor (aHCM) platform equipped with the CognitionWall system (Noldus Information Technology, The Netherlands) as previously described (Chen et al., 2022). Before conducting the CognitionWall assessments, mice were familiarized with the aHCM environment by individually housing for 3 days.

The CognitionWall has three entrances (left, middle, and right) placed in front of the food dispenser. In order to receive a reward of one food pellet (Dustless Precision Pellets, 20 mg, Rodent Grain-Based Diet, Bio-Serv, New Jersey), the mouse needs to enter the “correct” entrance five times (Fixed Ratio 5 schedule). The assessment of dynamic learning abilities encompasses a total duration of 96 h, divided into two phases of 48 h each: the initial learning phase and the reversal learning phase, as previously described in detail (Chen et al., 2022). During the initial learning phase, the left entrance of the CognitionWall is the “correct” entrance, while during the reversal learning phase, the right entrance is the “correct” entrance. The initial learning phase acts as a training phase, allowing the mice to learn the principle of the test (entering the “correct” entrance). The purpose of the reversal learning phase is to evaluate the learning flexibility of the mice, as they are required to “forget” their knowledge of the prior “correct” entrance and re-learn the new “correct” entrance. The PhenoTyper home-cages with the CognitionWall system is fully automated. Switching the monitoring of the “correct” entrance is programmed by the system at the end of the initial learning phase (48 h after the beginning of the assessment). In addition, the system automatically records the mouse’s behavior, using an infrared-sensitive video-based observation system located on the top unit of the aHCM. All the tracking data were acquired through the Etho-Vision XT software v14 (Noldus Information Technology, The Netherlands) and sampled at a rate of 15 fps. Raw data were uploaded to the web-based AHCODA-DB (Sylics, Bilt-hoven, The Netherlands) for meta-data processing and primary analysis.

### 2.4 Evaluation of learning process, patterns, and levels of learning ability of individual mouse

**Figure 1:**
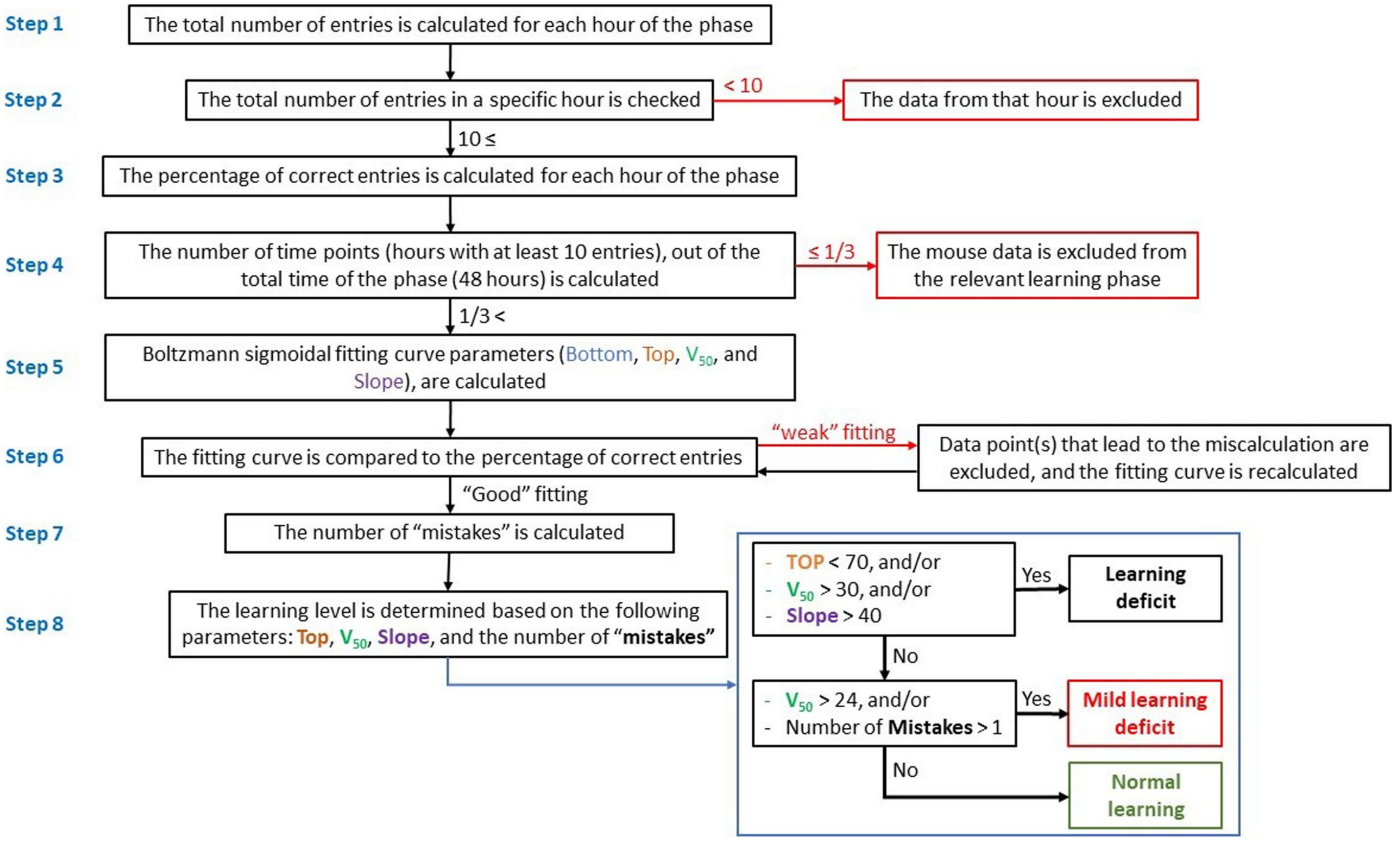
Flow-chart showing data processing and calculation of learning process and level. Evaluation of the learning process and calculation of the learning level are based on the hourly percentage of entries to the correct entrance (right entrance) during reversal learning, and calculation of a fitting curve.

In order to evaluate the learning process, pattern and learning ability of an individual mouse, we developed a new method for data processing as indicated in the flow-chart (Figure 1). This method used the hourly percentage of entries to the CognitionWall “correct” entrance to evaluate the learning process in each hour and then calculate a fitting curve that represents the learning pattern.

*Step 1*: the total number of entries into the CognitionWall was calculated hourly during the test. Since each mouse could choose the number of times to enter the CognitionWall during each hour, there were instances where a mouse did not enter at all or entered only a few times. A low number of hourly entries might indicate a lack of motivation in the mouse to obtain the reward and might not necessarily reflect its learning effort. Consequently, hours with a low entry rate were considered “noise” in the calculation of the fitting curve.

*Step 2*: to minimize “noise,” data from any hour with fewer than 10 total entries were excluded.

*Step 3*: the percentage of entries through the correct entrance (left entrance during the initial learning phase and right entrance during the reversal learning phase) was calculated for each test hour.

*Step 4*: the number of hours in which the mouse entered the CognitionWall at least 10 times was calculated and used to construct a fitting curve. Mice that entered the CognitionWall for less than one-third of the hours (fewer than 17 out of 48 h in each learning phase) were excluded, as a low number of hours would not permit an accurate calculation of the fitting curve and could impact the assessment of learning.

*Step 5*: a fitting curve for each mouse and each learning phase (initial & reversal) was calculated using the Boltzmann sigmoidal formula (Eq. 1):


(1) y=Bottom+Top−Bottom/1+e^V50−x/Slope

The fitting curves were calculated using the Prism software (GraphPad Software, La Jolla, CA), with the following parameters: x = the phase hour (1 to 48). Bottom = the low limit of the curve. This parameter represents the learning baseline. In the initial learning, the mouse started from “zero”; therefore, the bottom parameter was set to 0. In reversal learning, this parameter was set to be between 0 and 100. Top = the upper limit of the curve. This parameter represents the highest learning score (% of correct entries). This parameter can be between 0 and 100. V_50_ = the hour at which the percentage of correct entries is halfway between the Bottom and Top. Lower V_50_ reflects faster learning, while higher V_50_ reflects slower learning. Slope = the steepness of the curve, higher slope denotes a shallow curve.

**Figure 2:**
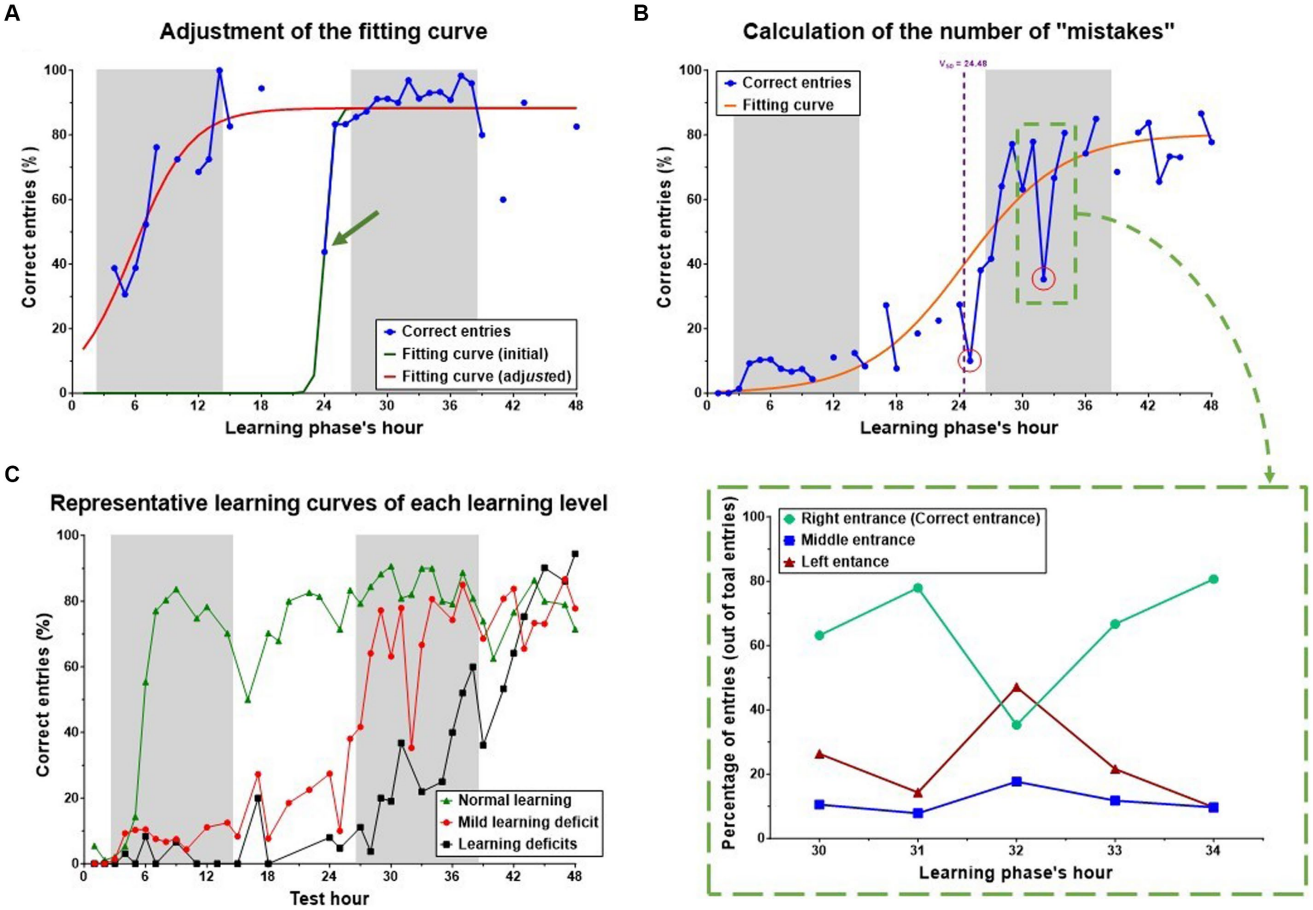
Adjustment of the fitting curve and calculation of the number of “mistakes.” **(A)** Representative example of adjustment of the fitting curve. The data from the 24th-hour timepoint led to a miscalculation of the fitting curve (green line). Exclusion of this timepoint (for the purpose of calculation of the fitting curve), results in a fitting curve that better represents the learning process (red line). Gaps in the correct entries curve resulted from excluding the data from hours with less than a total of 10 entries. **(B)** Representative example of the calculation of “mistakes.” “Mistake” was defined as a time point during the learning phase (after the V_50_), in which the percentage of correct entries was 40% or more below the fitting curve (red circles). **(C)** Representative learning curves of three mice. Each of the three mice represents one of the three learning levels: normal learning (green), Mild learning deficits (red), and learning deficits (black). Both mice that were defined as having Mild learning deficits (red) or learning deficits (black), showed a slow learning pattern, compared to the mouse that was defined as having normal learning (green). Shaded/unshaded regions in the graph represent the dark/light phase (respectively).

*Step 6*: the fitting curve was roughly compared to a scatter graph of the percentage of correct entries. In few cases, the percentage of correct entries at one of the time points (hour of the learning phase) caused a miscalculation of the fitting curve and resulted in a “weak” fitting (Figure 2A). In these cases, the data from that time point were excluded and the fitting curve was re-calculated. It is important to note that the data from that time point was excluded only for the purpose of calculating the fitting curve but were used for all other calculations (number of “mistakes,” etc.).

*Step 7*: the number of “mistakes” was calculated. “Mistake” was defined as a time point during the learning phase (after the V_50_), in which the percentage of correct entries was 40% or more below the fitting curve (Figure 2B).

*Step 8*: the learning level (for each learning phase) was determined based on the following parameters: Top, V_50_, Slope, and the number of mistakes. Three levels of learning were used, which represent the general learning ability/performance of each mouse: “Normal learning,” “Mild learning deficits,” and “Learning deficits” (Table 1).

**Table 1:** Criteria for each of the learning levels.

Learning Level	Reversal learning
Normal learning	70 ≤ Top and V_50_ ≤ 24 and Slope ≤ 40 and Number of mistakes ≤1
Mild learning deficits	70 ≤ Top and Slope ≤ 40 and 24 < V_50_ and/or 1 < number of mistakes
Learning deficits	Top <70 and/or 30 < V_50_ and/or 40 < Slope

### 2.5 Statistical analyses

All statistical analyses and calculations of fitting curves were conducted with the Prism software Version 10 (GraphPad Software, La Jolla, CA). The hourly distance moved, the total number of entries, and the percentage of entries were analyzed by two-way repeated-measures ANOVA or Mixed-effects model (REML) and Tukey’s multiple comparisons test. Data are expressed as mean values ± SEM.

## 3 Results

### 3.1 Assessment of activities of HEMI and NCAR mice and their exposure to LIB

As outlined in the Methods section, the CognitionWall test was employed to assess the learning levels and patterns of each individual mouse during the initial discrimination learning and the reversal learning phases. Prior to learning behavioral assessments, we evaluated each mouse’s locomotor activity by measuring hourly distance moved and the total number of entries to the CognitionWall during each hour of the test to ensure the cognitive ability measurement without the impact from impaired locomotor activity.

**Figure 3:**
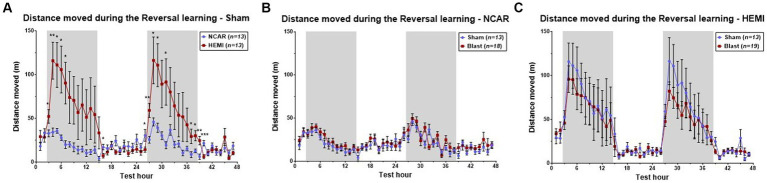
Hourly distance moved. **(A)** HEMI-Sham mice showed higher hourly moving distance during the dark phases, as compared to the NCAR-Sham mice. **(B,C)** While significant differences were found between the sham groups, no significant differences were found between the sham and blast groups of the NCAR **(B)** and the HEMI **(C)**. Shaded/unshaded regions in the graph represent the dark/light phase (respectively). Data is presented as Mean ± SEM. *p < 0.05, and **p < 0.01.

Both HEMI and NCAR Sham groups showed an increase in the distance moved during the dark phases as compared to the light phases. However, the HEMI group showed a significantly higher moving distance as compared to the NCAR group during the reversal learning phase as determined by Two-way RM ANOVA: [Time: *F*(1.862, 44.68) = 6.958, *p* < 0.0001; Group: *F*(1, 24) = 4.701, *p* = 0.0403; Time × Group: *F*(46, 1,104) = 3.856, *p* < 0.0001; Subject: *F*(24, 1,104) = 25.51, *p* < 0.0001] (Figure 3A).

Studies on effects of LIB exposure on activities of HEMI and NCAR groups were made by comparing hourly mean distance moved between the Sham and Blast groups (Figures 3B,C). Results indicated no significant differences between the NCAR Sham and Blast groups [Time: *F*(11.13, 322.9) = 9.284, *p* < 0.0001; Group: *F*(1, 29) = 1.071, *p* = 0.3092; Time × Group: *F*(46, 1,334) = 1.117, *p* = 0.2753; Subject: *F*(29, 1,334) = 12.94, *p* < 0.0001].

With the HEMI groups, although LIB appeared to induce a slight decrease in reversal learning during both dark phases, these differences did not reach statistical significance upon comparing between the HEMI Sham and Blast groups [Time: *F*(4.687, 103.2) = 39.35, *p* < 0.0001; Group: *F*(1, 30) = 0.2318, *p* = 0.6337; Time × Group: *F*(46, 1,380) = 0.4483, *p* = 0.9995; Subject: *F*(30,1,380) = 26.52, *p* < 0.0001].

### 3.2 Cognitive flexibility of the reversal learning

The main purpose of the reversal learning phase is to evaluate cognitive learning flexibility of the HEMI and NCAR mice. In the initial learning phase, mice learned that in order to receive a food pellet, they need to enter the left entrance of the CognitionWall for five times. In the reversal learning phase, mice need to “forget” their previous knowledge and re-learn to enter the correct (right) entrance to receive a food pellet. This process of relearning requires cognitive flexibility.

**Figure 4:**
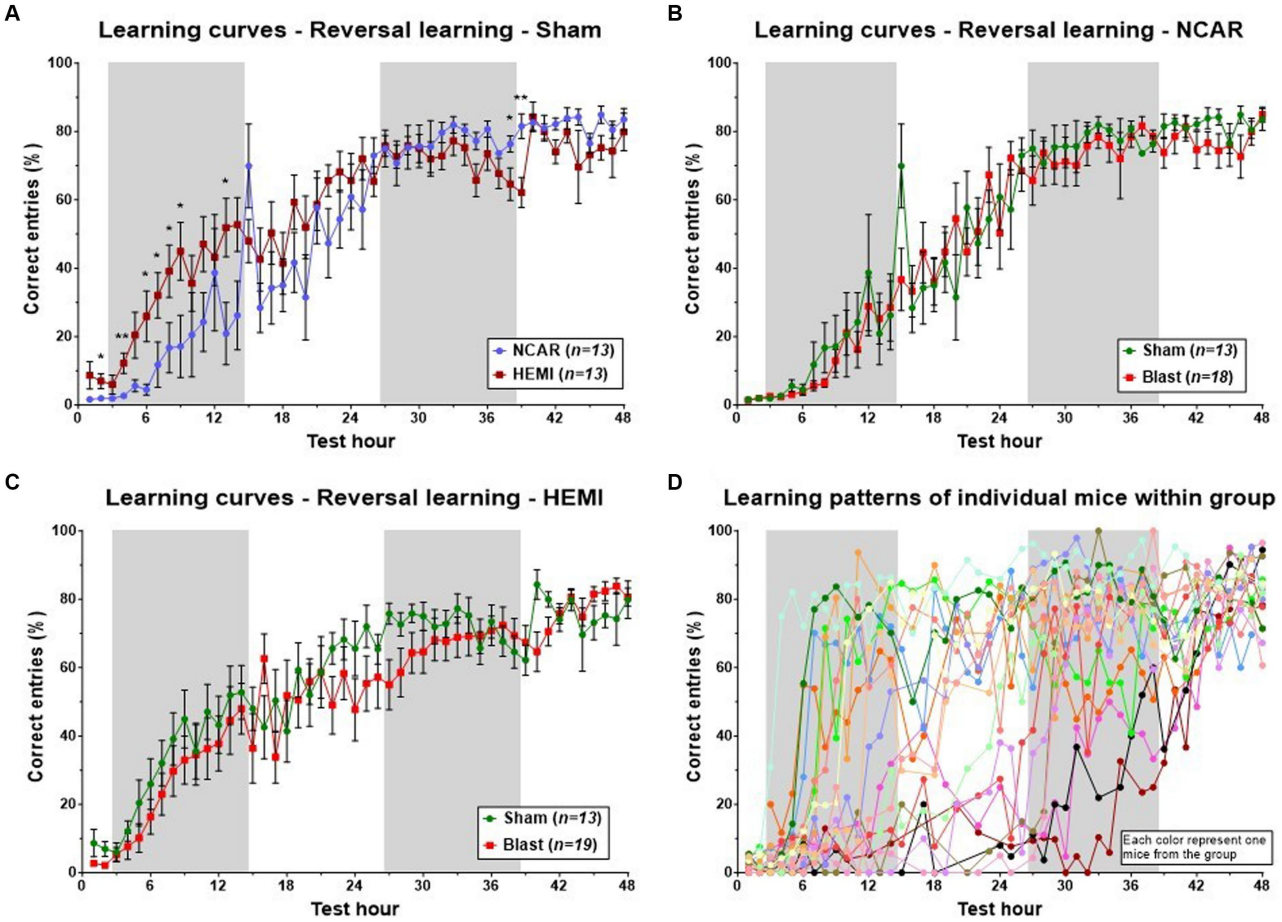
Learning patterns with the aHCM CognitionWall assessment at the group level. The learning pattern was evaluated based on the hourly percentage of correct entries to the CognitionWall test. **(A)** The HEMI mice showed a higher percentage of correct entries during the first 24 h of the phase, but lower during the last 10 h compared to the NCAR mice. **(B,C)** To evaluate the potential effect of exposure to blast on the mouse’s learning flexibility, the learning patterns, at the reversal learning phase, were compared between the sham and blast groups of each genotype separately. **(D)** Examination of the learning patterns of each mouse within one (representative) group reveals a complexity in the learning patterns that are not fully reflected when evaluating all the mice as a group (each color represents one mouse from the group, *n* = 19). While all the mice showed similar results at the beginning and end of the phase, they exhibited a wide variety in between. Shaded/unshaded regions in the graph represent the dark/light phase (respectively). Data is presented as Mean ± SEM. *p < 0.05, and **p < 0.01.

Evaluation of the learning process for HEMI and NCAR (sham) groups revealed a sigmoidal pattern, starting with a “baseline” plateau (about 0–10% of correct entries), followed by an increase in the percentage of correct entries during the first dark phase and the subsequent light phase, and finally culminating in a plateau of about 70–90%. Both groups exhibited a similar pattern and performance during the latter half of the learning phase (from the 25th to the 48th hours of the phase). However, during the initial light and dark phases, the HEMI group demonstrated a higher mean percentage of correct entries compared to the NCAR group [Time: *F*(3.575, 61.46) = 48.22, *p* < 0.0001; Group: *F*(1, 24) = 2.178, *p* = 0.1530; Time × Group: *F*(47, 808) = 2.395, *p* < 0.0001] (Figure 4A). The HEMI group continued to present a slightly higher percentage of correct entries during the second light phase. From the second dark phase until the end of the test, the NCAR group exhibited a similar or higher percentage of correct entries compared to the HEMI group. No significant differences in the hourly mean percentage of correct entries were found between the NCAR-Sham and NCAR-Blast groups [Time: *F*(2.803, 56.31) = 71.96, *p* < 0.0001; Group: *F*(1, 29) = 0.2070, *p* = 0.6525; Time × Group: *F*(47, 944) = 0.7200, *p* = 0.9214] (Figure 4B). However, during most of the reversal learning phase, the HEMI-Sham group showed a slightly higher (though not significant) mean percentage of correct entries compared to the HEMI-Blast group [Time: *F*(4.687, 103.2) = 39.35, *p* < 0.0001; Group: *F*(1, 30) = 1.311, *p* = 0.2612; Time × Group: *F*(47, 1,035) = 1.108, *p* = 0.2883] (Figure 4C).

Analysis of group-level learning patterns indicated significant fluctuations and variability within each of the four “two-by-two” groups, categorized by two factors: NCAR and HEMI, each subjected to either Sham or Blast exposure. A closer look at a representative group’s individual performances revealed marked differences among individual mouse (refer to Figure 4D). To facilitate a deeper understanding of these varied learning patterns and competencies, we developed a novel evaluation method to assess each mouse’s learning proficiency (detailed in the Method section’s flow chart, Figure 2). Utilizing this methodology, we classified each mouse into one of three distinct learning levels: Normal, Mild learning deficits, and Learning deficits. Notably, in this study, two mice from the NCAR-Sham group were excluded due to their inactivity—registering fewer than 10 entries per hour for over a third of the phase duration (as outlined in step 4, Figure 1).

### 3.3 Evaluation of the learning pattern and performance of individual mouse during the reversal learning phase

**Figure 5:**
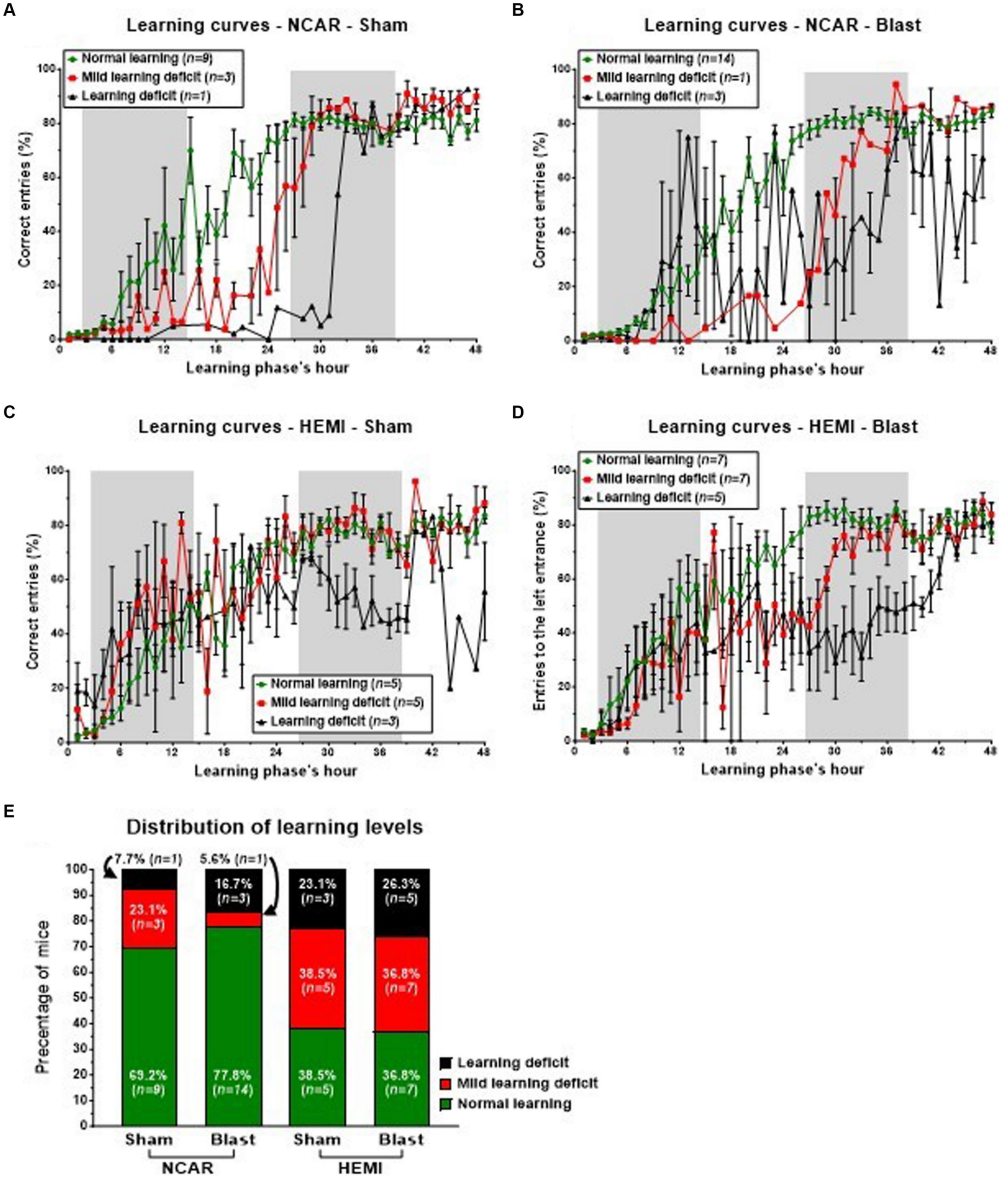
Learning patterns and distribution of mice to different learning levels in the reversal learning phase. The switch of the “correct” entrance (from the left one to the right one), challenged the mice and required two semi-connected processes: “forget” their previous knowledge of which is the “correct” entrance and to re-learn which is the “new” “correct” entrance. While the majority of NCAR mice showed a normal learning level **(E)**, the HEMI mice showed a much lower percentage of Normal learning level (no statistical analysis was performed due to the low sample size). The learning level represents a general level of learning and not a specific learning pattern. The high fluctuations and SEM in the subgroup of NCAR-Blast mice, which were defined as having learning deficits **(B)**, suggest that this subgroup includes some different patterns. Shaded/unshaded regions in the graph represent the dark/light phase (respectively). Data in **A–D** is present as Mean ± SEM (if applicable). Some error bars cannot be calculated/presented as the data at some time points is based on one mouse only.

In the initial hours of the phase, both Sham and Blast NCAR groups (as illustrated in Figures 5A,B, respectively) exhibited delayed learning, as indicated by a low percentage of ‘correct entries,’ in comparison to their HEMI counterparts (as shown in Figures 5C,D, respectively). Specifically, during the first 6 h and from the 33rd hour onward, all mice in the NCAR-Sham group maintained a consistent mean percentage of the correct entries (Figure 5A). However, between the 7th and 32nd hours, mice identified with Mild learning deficits (*n* = 3) demonstrated a lower mean percentage of correct entries compared to those with normal learning abilities (*n* = 9). Furthermore, the NCAR-Sham mice with Mild learning deficits exhibited a quicker learning pace than those with Learning deficits, indicating a prolonged period required for learning. Similarly, all NCAR-Blast mice displayed a consistent mean percentage of correct entries during the first 7 h of the reversal learning phase (Figure 5B).

During the 48-h reversal learning phase, HEMI-Sham mice with either Normal learning or Mild learning deficits exhibited comparable learning patterns, as depicted in Figure 5C. However, within this group, the mice identified with Mild learning deficits (*n* = 5) experienced significant performance fluctuations. Those classified with Learning deficits (*n* = 3) demonstrated notably poorer performance in the phase’s latter half. Similarly, HEMI-Blast mice maintained consistent learning patterns for the initial 20 h and the concluding 6 h. Nonetheless, mice within this group, regardless of being identified with Mild learning deficits or Learning deficits, displayed considerable performance variability (Figure 5D). HEMI-Blast mice with Normal learning (*n* = 7) and those with Mild learning deficits (*n* = 7) reached a comparable percentage of correct entries starting from the 38th hour. However, from the 8th to the 37th hour, mice with Mild learning deficits recorded a lower percentage of correct entries than their counterparts with Normal learning. Moreover, this subgroup exhibited delayed learning initiation, evident from a persistently low percentage of correct entries until the 26th hour. In contrast, mice with Learning deficits (*n* = 5) initially matched or exceeded the mean percentage of correct entries of mice with Normal learning during the phase’s first 13 h. Subsequently, from the 14th hour to the test’s conclusion, these mice’s mean percentages of correct entries declined, exhibiting greater variability compared to mice identified with Normal learning.

The distribution of mice into three learning levels highlighted differences between the groups, although no statistical analysis was conducted due to the small sample sizes (Figure 5E). Among the NCAR groups, 69.2% (*n* = 9) of NCAR-Sham and 77.8% (*n* = 14) of NCAR-Blast mice were categorized as having normal learning. In contrast, a significantly smaller proportion of the HEMI-Sham and HEMI-Blast groups were classified at 38.5% (*n* = 5) and 36.8% (*n* = 7), respectively. Furthermore, the incidence of Mild learning deficits or Learning deficits was notably higher in the HEMI groups compared to the NCAR groups. Specifically, in the NCAR-Sham and NCAR-Blast groups, 23.1% (*n* = 3) and 5.6% (*n* = 1), respectively, had Mild learning deficits, while 7.7% (*n* = 1) and 16.7% (*n* = 3) experienced Learning deficits. Conversely, in the HEMI-Sham and HEMI-Blast groups, a higher percentage of mice were affected by Mild learning deficits, 38.5% (*n* = 5) and 36.8% (*n* = 7) respectively, and Learning deficits, 23.1% (*n* = 3) and 26.3% (*n* = 5).

### 3.4 Different learning patterns within each learning level

**Figure 6:**
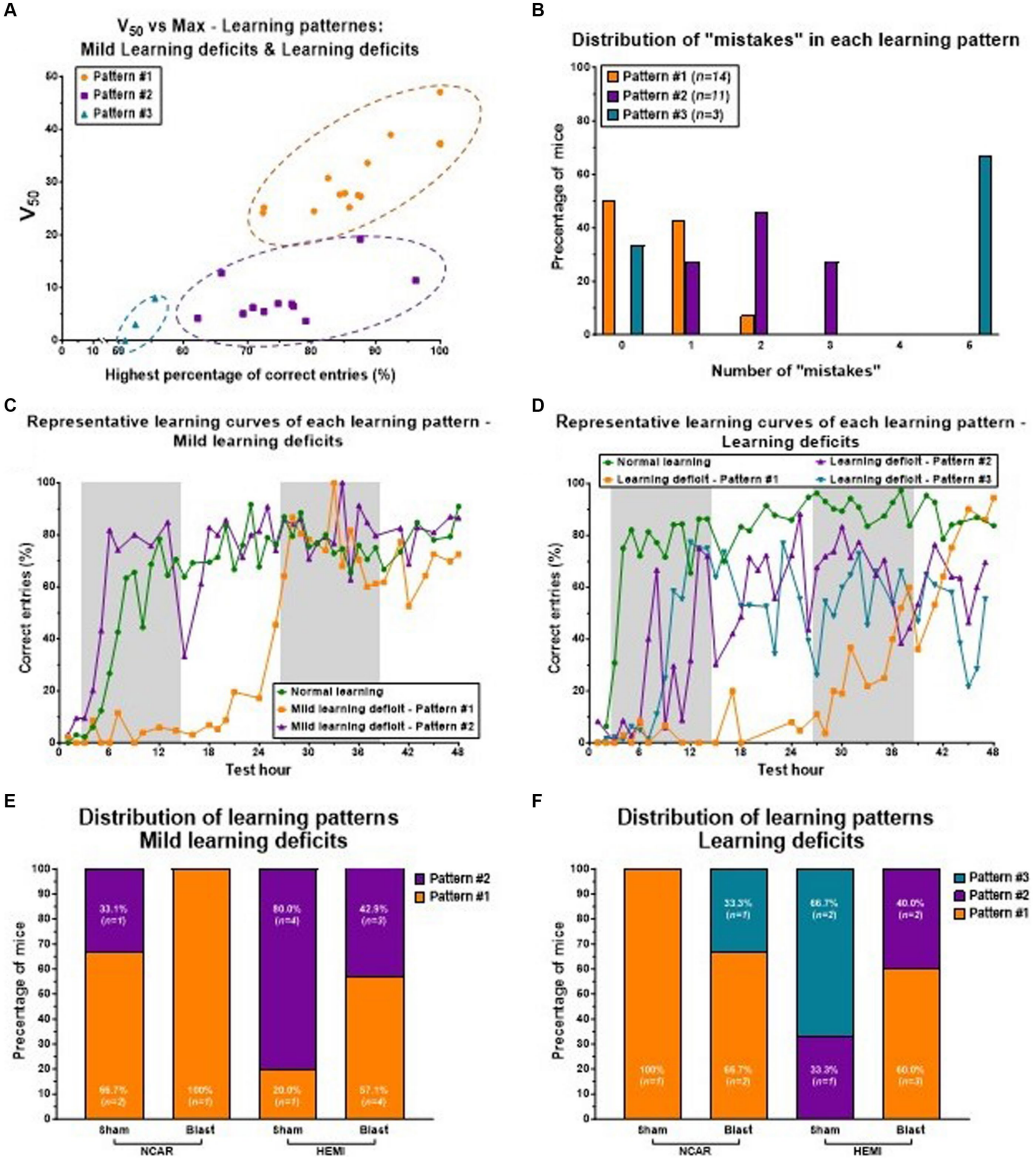
Learning patterns of Mild learning deficits and Learning deficits in mice. Mice having Mild learning deficits and Learning deficits both showed three learning patterns: **Pattern #1** to **Pattern #3**. **(A)** The distribution of three learning patterns is based on the two parameters of the learning curve: the highest percentage of correct entries (the Top parameter), and the V_50_ parameter. Mice with learning parameter #1 showed a high percentage of correct entries, but with a slow (delayed) learning rate (as represented by the V_50_ parameter). Mice with learning parameter #2 showed a mid-high percentage of correct entries, but with a fast-learning rate. Mice with learning parameter #3 showed a relatively low percentage of correct entries, but a fast-learning rate. **(B)** Mice with learning **Pattern #2** showed a higher number of “mistakes” compared to mice with learning **Pattern #1**. **(C,D)** representative learning curves of mice with Mild learning deficits **(C)** or Learning deficits **(D)**. Each line (color) represents different learning patterns. **(E,F)** The distribution of the different learning patterns in each of the groups. Mice with Mild learning deficits showed only **Patterns #1** or **#2 (E)**, while mice with Learning deficits showed all three learning patterns **(F)**. Shaded/unshaded regions in the graph **(C,D)** represent the dark and light phases, respectively.

Analysis of the learning level subgroups showed both fluctuations and relatively high variability, particularly within the Mild learning deficit and Learning deficit categories. This observation indicates that despite belonging to the same learning level, individual mice may exhibit distinct learning patterns. By comparing each mouse’s learning curve from both Mild learning deficit and Learning deficit subgroups with those of the normal learning subgroup within the same category, we identified three distinct learning patterns (illustrated in Figures 6A,B):

**Pattern #1:** this pattern is defined by a noticeable delay in learning onset (indicated by a high V_50_ value) compared to mice classified with normal learning abilities.

**Pattern #2:** here, mice show learning performances that are either similar to or slightly lower than those of mice with normal learning, in terms of the percentage of correct entries.

**Pattern #3:** mice in this category match the performance of mice with normal learning during the initial hours of the reversal learning phase, but this is followed by significant performance fluctuations and a generally lower percentage of correct entries compared to mice with normal learning.

Notably, mice demonstrating **Pattern #2** were observed to make more errors than those exhibiting **Pattern #1** (see Figure 6B). Representative learning curves showcasing these distinct patterns are displayed for mice with Mild learning deficits (Figure 6C) and for those with significant deficits (Figure 6D), with each line (or color) denoting a different learning pattern.

The analysis of the learning pattern distribution highlights the potential influence of genotype and exposure to LIB on learning levels. In the group of mice with Mild learning deficits (referenced in Figure 6E), a majority of the NCAR-Sham mice displayed learning **Pattern #1** (66.7%, *n* = 2), whereas a significant portion of HEMI-Sham mice exhibited learning **Pattern #2** (80%, *n* = 4). For both genotypes, exposure to blast appeared to increase the proportion of mice demonstrating learning **Pattern #1**, with all NCAR-Blast mice (100%, *n* = 1) and more than half of the HEMI-Blast mice (57.1%, *n* = 4) showing this pattern. Notably, no mice with Mild learning deficits were observed to exhibit learning **Pattern #3**. Conversely, mice classified with Learning deficits (illustrated in Figure 6F) presented all three identified learning patterns. The sole NCAR-Sham mouse in this category showed learning **Pattern #1** (100%, *n* = 1), while HEMI-Sham mice were divided between **Pattern #2** (33.3%, *n* = 1) and **Pattern #3** (66.7%, *n* = 2). The influence of blast exposure on these mice mirrored the trend seen in those with Mild learning deficits, with two-thirds of NCAR-Blast mice (66.7%) displaying learning **Pattern #1** and one mouse (33.3%) showing **Pattern #3**. Among the HEMI-Blast mice, the majority were categorized under learning **Pattern #1** (60%, *n* = 3), with the remainder exhibiting learning **Pattern #2** (40%, *n* = 2).

### 3.5 Evaluation of mouse’s cognitive learning flexibility

**Figure 7:**
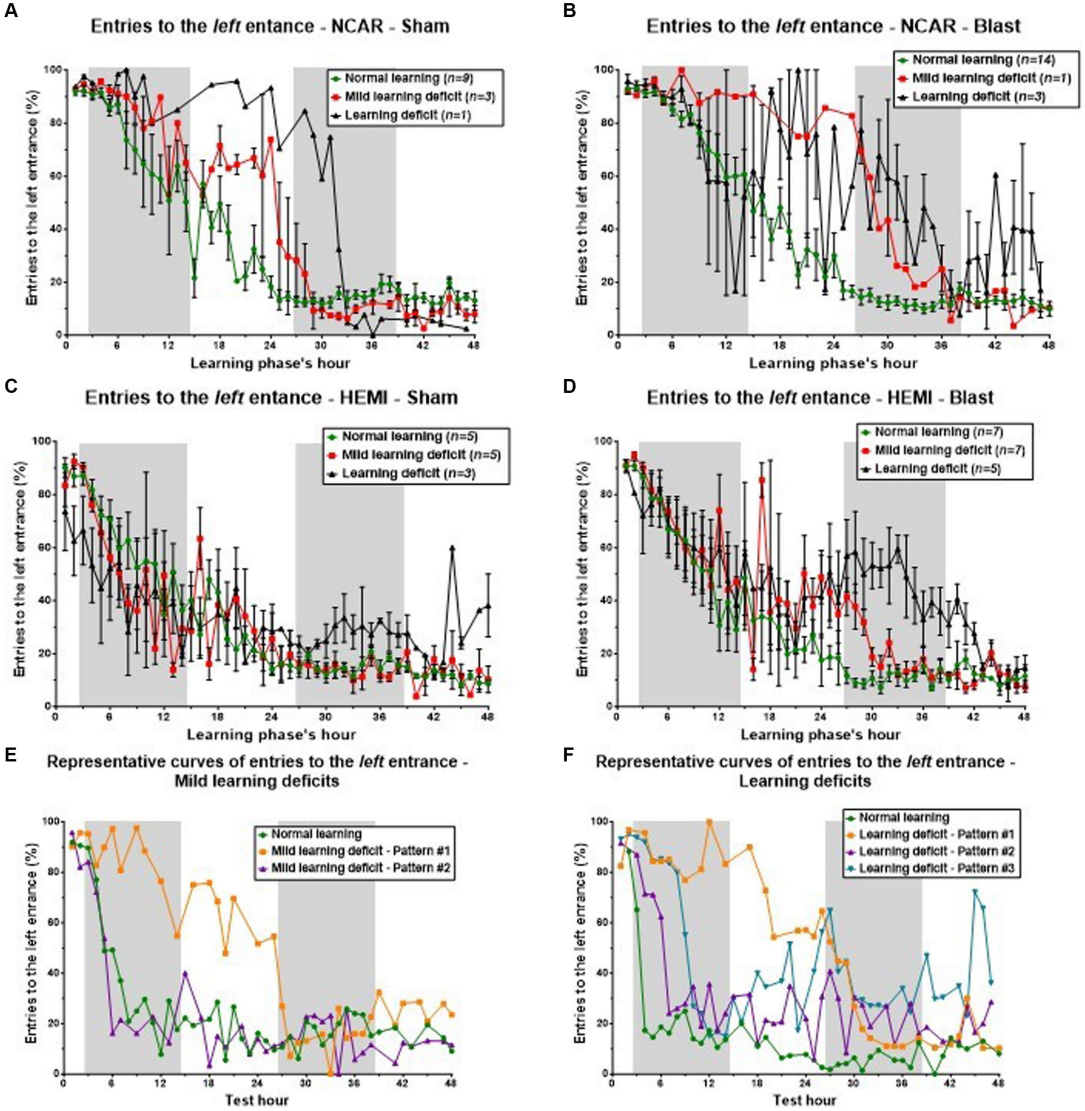
Cognitive learning flexibility. Reduced cognitive learning flexibility is one of the factors that might lead to learning deficits. The purpose of the reversal learning phase is to evaluate that “flexibility,” as the mouse is required to “switch” from the left entrance to the right one. In this process of “switching,” the percentage of entries to the left entrance (considered as perseverative errors) is expected to be reduced, while the percentage of entries to the right entrance is expected to increase. The errors to the middle entry are considered as neutral errors. Therefore, an examination of the number of entries to the left entrance can give important information on the mouse’s cognitive learning flexibility. To evaluate the connection between learning flexibility and learning level, we examined the mice’s hourly percentage of entries to the left entrance during the reversal learning phase. The results were gathered according to the mouse’s genotype, exposure group (Sham or Blast), and learning level as shown in Figure 3 [i.e., mice in the NCAR-Sham normal learning (Figure 4A) are the same mice as in the NCAR-Sham normal learning subgroup in Figure 3A]. **(A–D)** The hourly percentage of entries to the left entrance. Mice defined as having Mild learning deficits or learning deficits showed a slower and/or delayed reduction in the percentage of entries to the left entrance. However, the high fluctuation and SEM (in all subgroups) imply that additional factors may influence the learning levels. **(E,F)** The hourly percentage of entries to the left entrance according to the learning level: Mild learning deficit and Learning deficits (**E,F**, respectively). Mice in learning **Pattern #1** showed a clear delay in the switching from the left entrance to the right one, supporting the hypothesis that a reduction in learning flexibility may have affected the mice’s learning level (especially learning **Pattern #1**). Shaded/unshaded regions in the graph represent the dark/light phase (respectively). Data is present as Mean ± SEM (if applicable). Some error bars cannot be calculated/presented as the data at some time points are based on one mouse only.

One key facet of cognitive learning flexibility is the capacity to discard old knowledge in favor of acquiring new information. During the initial learning phase, mice were trained to use the left entrance of the CognitionWall. Conversely, in the reversal learning phase, they were required to switch their preference to the right entrance. We propose that a deficit in learning may stem from limited cognitive learning flexibility, manifested as a delay in transitioning from the left to the right entrance. To examine this theory, we monitored the hourly percentage of entries into the left entrance as an indicator of the mice’s ability to “forget” their prior learning. This metric also helped us determine whether the observed differences among the three learning levels and patterns could be attributed to variations in the mice’s capacity to discard old knowledge. Our findings reveal that, by the end of the reversal learning phase, there was a universal decline in left entrance usage across all mice, indicating some degree of learning and adaptation (see Figure 7).

Initially, all NCAR-Sham mice displayed a comparable frequency of left entrance usage during the early hours of the phase (Figure 7A). Subsequently, mice with normal learning capabilities exhibited a steady and swift decrease in using the left entrance, indicating effective relearning and entrance switching. In contrast, mice with Mild learning deficits or Learning deficits required additional time to adjust and adopt the new entrance preference. This divergence in learning speeds and abilities among the groups—Normal learning, Mild learning deficits, and Learning deficits—became particularly evident in the middle of the assessment phase, despite all mice showing similar entry patterns at both the start and conclusion of the phase.

In the analysis of NCAR-Blast mice (referenced in Figure 7B), those with either Normal learning abilities or Mild learning deficits exhibited patterns similar to their NCAR-Sham counterparts. Yet, NCAR-Blast mice with Learning deficits maintained a comparable frequency of left entries to that of NCAR-Blast mice with Normal learning abilities until the conclusion of the first dark phase. From the 9th hour onward, these mice displayed considerable variability in their behavior. After the first dark phase, there was an increase in the mean percentage of left entrance entries among these mice, even though their variability remained notably high.

For HEMI-Sham mice (Figure 7C), those with Normal learning abilities or Mild learning deficits showed consistent behavior throughout the phase. In the phase’s first half, HEMI-Sham mice with Learning deficits used the left entrance less frequently than those with Normal learning abilities. In the latter half, however, their average entry rate to the left entrance increased, surpassing that of other mice.

HEMI-Blast mice across all three learning levels initially demonstrated a uniform mean entry rate to the left entrance during the first 12 h and in the closing hours of the phase (Figure 7D). During the intervening period, mice with Normal learning abilities consistently reduced their usage of the left entrance. In contrast, mice with either Mild learning deficits or Learning deficits exhibited an increase in the average percentage of left entrance entries, accompanied by a high degree of variability within each subgroup. Mice exhibiting learning **Pattern #1**, as shown in Figures 7E,F, demonstrated a pronounced delay in switching from the left to the right entrance. This observation supports the hypothesis that decreased learning flexibility adversely impacts the mice’s learning performance, particularly in learning **Pattern #1**.

## 4 Discussion

Memory and learning deficits are commonly reported in individuals with blast-induced mTBI (Bogdanova and Verfaellie, 2012; Cook et al., 2014; Karr et al., 2014; Miller et al., 2017; Pagulayan et al., 2018). However, assessing these impairments in humans is challenging due to methodological limitations and conceptual misunderstandings. Moreover, outdated notions, such as the idea that each type of clinical dementia corresponds to a distinct brain pathology, may hinder our comprehension of LIB-induced brain pathologies (Dams-O’Connor et al., 2023; Rost, n.d.). The assessment of learning and memory capabilities is a standard clinical practice for diagnosing and conducting research across various patient groups and age ranges. Within each group, a wide range of learning and memory abilities is typically observed (Uttl, 2005). Indeed, numerous studies employing sophisticated analytical techniques have identified distinct learning patterns (Uttl, 2005; Barbot et al., 2016; Padhy et al., 2016; Sáiz Manzanares et al., 2017; Breton et al., 2021; Lou et al., 2022). Given these findings, it is reasonable to conclude that different learning patterns may be indicative of varied types of damage affecting specific brain regions. Consequently, the nature and severity of disabilities among individuals may vary based on the extent and location of brain damage (Huang et al., 2017).

Pathological forms of tau phosphorylation have been identified as playing a significant role in neurodegenerative diseases, leading to reduced learning capacity and memory deficits, as seen in tauopathies like CTE and FTD (Morrison et al., 2022; Stathas et al., 2022; Stoner et al., 2023). Research has demonstrated that rTg450 HEMI mice that overexpress human tau exhibit frontal brain pathologies characteristic of FTD. These pathologies include synapse loss, increase in hyperphosphorylated tau, tau-containing neurofibrillary tangles (NFTs), β-amyloid (Aβ) accumulation, and upregulation of microglia and inflammatory indices, thereby making them an effective model for FTD research (Frautschy and Cole, 2010; Wes et al., 2014; Logan et al., 2018; de Oliveira et al., 2022). A recent study (Corrigan et al., 2021) highlighted tau hyperphosphorylation’s crucial role in cognitive and neurological deficits induced by blast exposure. In our study using the CognitionWall test, HEMI mice showed a significant increase in activity compared to NCAR mice (Figure 3A), aligning with hyperactivity observed in tau-mediated neuropathology development. The HEMI genotype, expressing the P301L mutant variant of human four-repeat Tau (4R0N tau_P301L_) predominantly in the forebrain, demonstrated Tau protein expression levels 13-fold higher than those in NCAR mice. This expression difference clearly leads to behavioral, histological, and functional disparities between the two genotypes (Kopeikina et al., 2013). While some pathologies are age-independent, others, such as the progression of neurodegenerative tauopathies are age-dependent (Ramsden et al., 2005; Santacruz et al., 2005; Yue et al., 2011; Logan et al., 2019). Studies have shown that the accumulation of tau pathology in rTg4510 transgenic mice, correlating with age-dependent memory declines, becomes evident at 6–8 months of age (Santacruz et al., 2005; Castanho et al., 2020). In our recent study (Jackson et al., 2024b), discrimination learning was assessed using the CognitionWall test in rTg4510 mice at 30 days following a single LIB exposure over a 48-h period. Results showed that blast-exposed rTg4510 mice showed a lower learning index compared with all other groups. The individual analysis identified either top ten increased or decreased expression of phosphopeptides in turquoise and black module eigenpeptides, characterized by such altered phosphopeptides associated with learning ability, that confer an increased risk for learning deficits following LIB exposure. In addition to the high expression levels of Tau, upregulation of microglia and neuroinflammation are also reported in rTg4510 mice (Wes et al., 2014; de Oliveira et al., 2022).

However, despite of physical differences between the HEMI and NCAR groups, neither genotype demonstrated obvious alterations in activities upon exposure to LIB. This absence of a marked difference may be due to the assessment method and the use of younger mice (2-month-old) in the current study.

The learning process evaluated through the CognitionWall test is continuous, unlike other learning assessments, such as the Morris Water Maze (MWM) and Barnes Maze, that are session-based. Studies have analyzed learning performance based on 24-h blocks for each phase of the test and/or the time it takes for mice to achieve a performance threshold (for example, selecting the “correct” entrance in 80% of the last 30 entries) (Hadas et al., 2016; Logan et al., 2018; Chen et al., 2022). However, these methods do not fully capture the dynamic nature of the learning process. A mouse might meet the 80% threshold at one point but subsequently drop to only 50%. To thoroughly assess the learning process, patterns, and abilities of each mouse, we analyzed the hourly percentage of entries to the “correct” entrance of the CognitionWall throughout the reversal learning phase. This approach mirrors the Learning Index [(Correct entries – Incorrect entries)/Total Entries] utilized by Logan et al. (2018). By evaluating the percentage of correct entries in one-hour segments, we can compare the performance of different mice across various hours of the phase with high resolution, shedding light on the learning dynamics.

The CognitionWall test offers the advantage of assessing individual mice over an extended period. This study, analyzing reversal learning patterns based on the hourly percentage of correct entries, revealed significant differences between HEMI and NCAR mouse groups in the first 24 h of the phase. However, a deeper examination of individual mouse within each group exposed a complexity in learning patterns not apparent when evaluating mice as a collective (Figure 4D). This heterogeneity was present in both HEMI and NCAR groups, irrespective of sham or LIB exposure. Consequently, we developed a new method employing the Boltzmann fitting curve, based on hourly data, to classify each mouse into one of three distinct learning levels: Normal learning, Mild learning deficits, and Learning deficits (see Figure 1). This detailed analysis highlighted differences between HEMI and NCAR mice, and variations stemming from LIB exposure (Figure 5E). Comparing the learning curves of mice with Mild learning deficits or Learning deficits to those with relatively Normal learning identified three distinct learning levels. Mice with Mild learning deficits presented normal-like performance during the beginning and the end of the learning period. All mice (regardless of their learning level) start the learning process from the same level, and therefore, showed similar results in the beginning of the learning period. At the end of the learning period, mice with Mild learning deficits showed high percentage of learning performance (similar to mice with normal learning), although theirs learning process was longer and/or required more trials (compare to mice with normal learning). Therefore, the difference between their performance and the performance of mice with normal learning, could mostly be seen during the “middle” of the learning period. Similar results were previously reported by Zuckerman et al. (2017). These findings emphasize the complexity of the learning process, suggesting that individual mice may exhibit unique brain deficits. Importantly, the analysis revealed that some sham mice also showed learning deficits and varied learning patterns, highlighting the complexity and diversity within each group. Moreover, this finding demonstrates the common assumption that the “control” group representing “normal” and hemogenic behavior properties (including learning abilities) is not evidence-based. Variations in learning ability, including learning deficits, can be found in any random group of animals or humans.

After initial categorization, subgroups were further divided into three distinct learning patterns, as depicted in Figure 6A. The results revealed that, within a subgroup sharing the same learning level, mice could exhibit varied learning patterns (Figures 6E,F). This diversity not only underscores the complexity within the population but also suggests that individual mouse may possess unique pathologies related to learning. The variation in learning pattern occurrence between the blast and sham groups, within each genotype, highlights several points: (1) genotypic differences influence the prevalence of each learning pattern, as evidenced by comparisons within sham groups. (2) Blast exposure may induce distinct pathologies. (3) Each genotype exhibits susceptibility to different types of pathologies, resulting in varied learning patterns. Learning **Patterns #1** and **#2** were characterized by some deficits, such as delayed learning or a reduced percentage of correct entries. However, in the final hours of the phase, their performance converged with that of mice demonstrating normal learning. In contrast, **Pattern #3** represented a more complex deficit, with mice initially showing normal learning behavior but failing to elevate the percentage of correct entries above 60%.

Motor activity analysis across the patterns also revealed differences. Mice in **Pattern #1** generally moved less, particularly in the blast-exposed groups (NCAR-Blast and HEMI-Blast) and made fewer visits to all three entrances of the CognitionWall, compared to those with normal learning. Mice exhibiting **Pattern #2** moved distances comparable to, or slightly greater than, those with normal learning, and they visited the CognitionWall more frequently, especially the HEMI mice during the dark phases. Conversely, mice with **Pattern #3** exhibited significantly higher activity levels, particularly in dark phases, traveling longer distances and making more visits to the CognitionWall than mice identified with normal learning. This heightened activity, along with fluctuations in activity levels, suggests impulsivity, potentially explaining these mice’s performance. Early-phase multiple visits to the CognitionWall may facilitate learning the correct entrance. However, this impulsivity may hinder the mice’s ability to consistently choose the correct entrance in later stages. Hadas et al. (2016) reported similar observations in rats exposed to dynamic sensory stimuli during development, identifying higher activity levels and learning rates in adulthood but poor performance in the presence of distractions. Despite uniform environmental stimuli exposure in our study (except for the blast, conducted under anesthesia), the observed high activity level might also indicate impulsivity.

Cognitive learning flexibility encompasses more than just acquiring new knowledge; it involves the replacement of old knowledge with new information through a sequential process. This progression involves several steps: (1) The mouse must recognize that entering the left entrance of the CognitionWall (representing “old” knowledge) is no longer rewarding. (2) The mouse must learn that the right entrance now offers rewards (“new” knowledge). (3) The mouse must integrate these insights, decreasing its visits to the unrewarding left entrance while increasing visits to the rewarding right entrance. The observed decrease in left entrance visits and corresponding increase in right entrance visits across all mice demonstrate successful navigation of the first two steps. However, while some mice quickly mastered all three steps, showcasing Normal learning, others faced challenges, indicative of Mild learning deficits or more pronounced learning difficulties. Classifying these mice into three distinct learning levels illuminates various factors influencing their ability to complete the entire learning process. The significant variability observed within each learning level suggests that the challenges in learning are multifaceted, rather than stemming from a single cause.

## 5 Limitations

The CognitionWall test facilitates the assessment of dynamic learning processes over an extended period. This test leverages the mouse’s natural motivation and activity patterns, making comparisons of learning processes across different mice challenging. Additionally, the prolonged isolation periods during the test could affect their behavior, given that mice are social animals. Despite these challenges, the data presented in this study effectively illustrate the complex nature of ‘real-world’ learning deficits resulting from blast-induced mTBI. The increased awareness for sex-related differences on the physiology, behavior and other aspects, has led to the increasing demand to include both males and females in each study. Even though, only males were used in this study. The decision to use males only, resulted from the novelty of this analysis method, and our effort to reduce the number of variables. Future studies with this novel method, which include females, will extend our understanding on sex-related learning behavior and learning deficits. Despite efforts to replicate the ‘real-world’ conditions of blast-induced mTBI and its impacts on behavior and learning, certain aspects of this study fall short of fully emulating the actual scenario due to various limitations. For instance, differences in brain size, structure, and orientation between mice and humans may influence the applicability of findings. The severity of learning deficits might also vary with age and the time elapsed post-injury, potentially intensifying with increased age and/or longer durations post-injury. Exposure to single versus repeated LIB, may also affect the incidence and level of learning deficits. Additionally, the small number of mice in certain learning levels and patterns constrained our capacity to thoroughly investigate the mechanisms underlying the development of these deficits. Comparisons between results from clinical and preclinical studies reveal significant differences. Primarily, most clinical assessments involve linguistic learning tasks, which are inapplicable to animal models. Furthermore, human participants are typically aware of being evaluated and understand the purpose and rules of the test, often conducted in familiar and safe settings. In contrast, assessments of animal learning and memory typically proceed without the animals’ awareness of the evaluation, lacking initial understanding of the test’s rules and objectives. Moreover, while it is common in preclinical studies to evaluate and report results at multiple time points throughout the test, many clinical studies do not provide or report data on the learning process in such detail.

## 6 Conclusion

This study has revealed a considerable degree of complexity and heterogeneity in learning flexibility across different mouse groups, notably in relation to LIB exposure. By employing the innovative CognitionWall system, we were able to categorize each mouse based on its specific learning level and pattern. The observed increase in learning deficits within the HEMI group, relative to the NCAR group, could be attributed to elevated tau expression, a factor known to contribute to neuropathology development. Interestingly, LIB exposure in NCAR mice resulted in a decreased incidence of Mild learning deficits but an increased prevalence of Learning deficits. Our findings suggest that various genotypes may exhibit distinct responses in learning ability post-LIB exposure, a revelation with potential implications in the clinical setting. Specifically, it emphasizes the need for behavioral and cognitive assessments of mTBI to consider diverse patient demographics, including ethnicity, sex, age, and injury type.

The identification of three unique learning patterns further indicates that Learning deficits may stem from multiple mechanisms. In summary, these results highlight the critical need for analyzing data at the level of individual animals and adopting a high-resolution approach to data evaluation. Such detailed analysis not only mirrors the individualized assessment common in clinical practice but also enhances the study’s applicability to translational research. By analyzing behavior at the individual level, this study paves the way for linking specific behavioral deficits with underlying cellular and molecular mechanisms, thereby increasing the relevance of our findings to translational studies.

## Data availability statement

The raw data supporting the conclusions of this article will be made available by the authors, without undue reservation.

## Ethics statement

The animal study was approved by Animal Care and Use Committee (ACUC) at the University of Missouri. The study was conducted in accordance with the local legislation and institutional requirements.

## Author contributions

AZ: Data curation, Formal analysis, Investigation, Methodology, Validation, Writing – original draft, Writing – review & editing. HS: Data curation, Formal analysis, Validation, Writing – review & editing, Investigation. AB: Data curation, Formal analysis, Investigation, Validation, Writing – review & editing. RL: Writing – review & editing, Investigation. GS: Writing – review & editing. DC: Writing – review & editing. IC: Writing – review & editing. JC: Conceptualization, Data curation, Investigation, Methodology, Validation, Writing – review & editing, Formal analysis, Project administration, Resources. ZG: Conceptualization, Data curation, Formal analysis, Funding acquisition, Investigation, Methodology, Project administration, Resources, Supervision, Validation, Writing – original draft, Writing – review & editing.
